# Assisted Reproductive Technology (ART): A Ray of Hope for Adolescent Idiopathic Premature Ovarian Failure

**DOI:** 10.7759/cureus.40723

**Published:** 2023-06-21

**Authors:** Nihar R Bhoi, Vipin Chandra, Charu Johari, Kshitiz Murdia

**Affiliations:** 1 Reproductive Medicine, Indira IVF Hospital Private Limited, Udaipur, IND; 2 Clinical Research and Operations, Indira IVF Hospital Private Limited, Udaipur, IND; 3 Reproductive Medicine, Indira IVF Hospital Private Limited, Alwar, IND

**Keywords:** assisted reproductive technology (art), hormone replacement therapy, adolescent, premature ovarian failure (pof), in vitro fertilization (ivf)

## Abstract

Adolescent idiopathic premature ovarian failure (POF) is extremely rare. In such conditions, there is a defect in the process of the folliculogenesis axis leading to a hypoestrogenic state and infertility. This disorder significantly impacts a woman's prospective health, fertility, and motherhood. A case of a 24-year-old female diagnosed with adolescent idiopathic premature ovarian failure under hormone replacement therapy who visited us for fertility management is discussed here. The couple underwent in vitro fertilization (IVF) using donor oocytes resulting in a twin pregnancy. The clinical significance of premature ovarian failure and subsequent reproductive guidance is highlighted in this case report.

## Introduction

Premature ovarian insufficiency is the cessation of menses before age 40. The average age at which menopause happens in the Indian population is between 40 and 50 years [1]. The incidence is estimated at around one in 10,000 in women less than 30 years [2]. The consulting physician should discuss comorbidities such as decreased bone mineral density, cardiovascular risks, sexual problems associated with primary ovarian failure, and fertility aspects with the patient and family [3]. The resumption of fertility is very unpredictable. In vitro fertilization (IVF) with donor oocytes give good results and should be offered in such a scenario.

## Case presentation

This is a case report of a married female diagnosed with premature ovarian failure during puberty who visited our unit for fertility management in her 20s.

**Figure 1:**
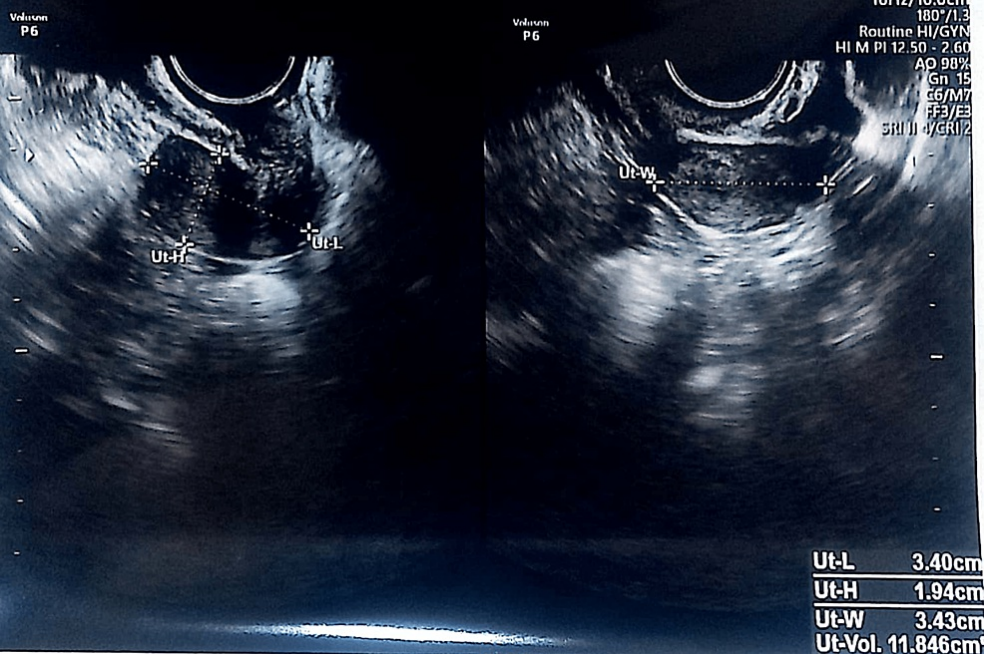
Ultrasound of the uterus

At her documented first visit, breast development was Tanner stage 1, pubic hair was Tanner stage 1, and axillary hair was absent. Serum testosterone, dehydroepiandrosterone, and serum prolactin were normal. The serum E2 value was 20 pg/mL, and anti-thyroid peroxidase (TPO) antibodies were absent. Her karyotyping was 46xx, and her ultrasound showed a uterus measuring 3.40 × 1.94 × 3.43 cm with bilateral streak ovaries (Figure 1). Her serum follicle-stimulating hormone (FSH) was 34.13 IU/L, anti-Mullerian hormone was <0.05 ng/mL, and serum luteinizing hormone (LH) was 12 IU/dL. At the present visit, the patient was phenotypically average, with a height of 168 cm, body mass index of 22.4 kg/m^2^, breast Tanner stage 4, pubic hair Tanner stage 4, and axillary hair present. The marriage was nonconsanguineous. In pedigree evaluation, neither congenital disability nor genetic disorder was found in the family. She had no history of ovarian surgery, radiation, or medications. The husband's semen parameters were in the normal range.

**Figure 2:**
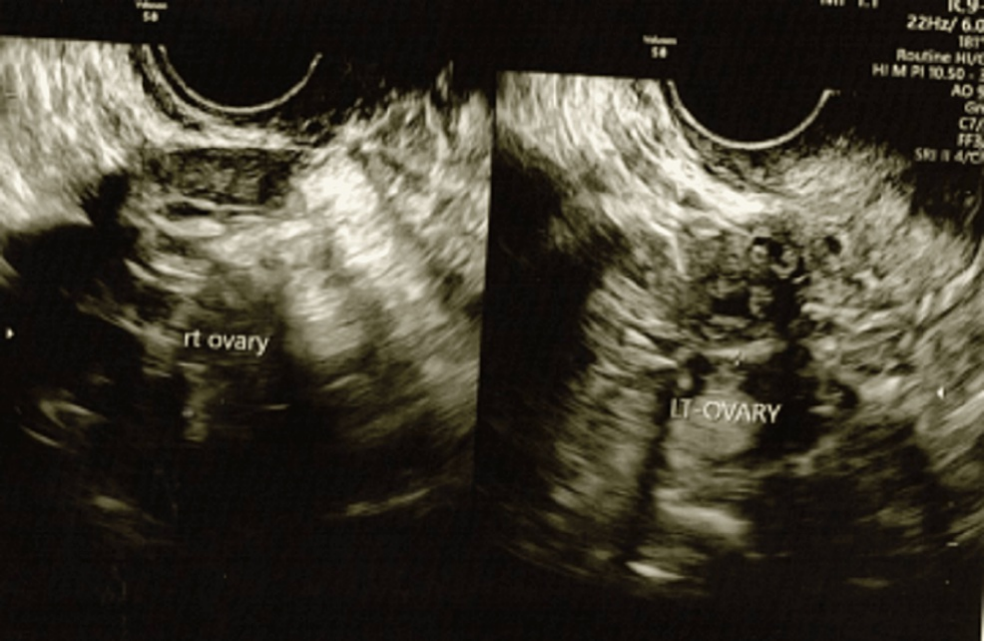
Ultrasound of the ovaries

The patient was started on a combined oral contraceptive pill with the intention of enhancing the uterine size. After the completion of three cycles of contraceptive pills, the uterus was measuring 6.0 × 2.8 × 5.3 cm in size with a homogenous endometrium of 5.1 mm in thickness. Bilateral ovaries were atrophic with no follicular activity (Figure 2). Thyroid and prolactin hormones were normal, and the anti-Mullerian hormone was <0.01 ng/mL.

The patient was counseled, and the need for donor oocytes was explained to the patient. The couple agreed to IVF with donor oocytes. A suitable donor was selected and screened before stimulation. The donor undergoes genetic (thalassemia) and infectious disease (human immunodeficiency virus (HIV), hepatitis C/hepatitis B surface antigen (HBsAg), and venereal disease research laboratory test (VDRL)) screening. The gonadotropin-releasing hormone (GnRH) antagonist protocol was used for stimulation. Intracytoplasmic sperm injection (ICSI) was done using the husband's sperm. Estradiol valerate 2 mg TDS was used for the endometrial preparation of the patient. A total of two embryos were transferred (grade 5AA and 5AA ) on the blastocyst stage after laser-assisted hatching. Serum β-human chorionic gonadotropin hormone value came after the 14th day of embryo transfer. Transvaginal ultrasound done at six weeks gestational age showed a dichorionic diamniotic twin pregnancy. Nuchal translucency at 12 weeks gestation was 2.4 mm and 2.2 mm for both fetuses, respectively. Aneuploidy screening at 13 weeks with a noninvasive prenatal screening test was normal. She received regular consultations from a clinical psychologist as and when required. The patient delivered by lower segment cesarean section (LSCS) healthy twins at 36 weeks of gestation.

## Discussion

Premature ovarian failure is defined as amenorrhea associated with ≥4 months of high gonadotropic hypogonadism in females younger than 40 years who have not undergone surgery, radiation, or medications that may impair ovarian function [3]. The presentation of premature ovarian failure may vary from oligomenorrhea to primary amenorrhea, to secondary amenorrhea.

A study identified 17 patients with non-chromosomal, non-iatrogenic premature ovarian failure in the adolescent population that were poorly characterized, and its incidence was unknown. It can also be caused by the disturbance of a previously established menstrual cycle as the reported mean age of diagnosis was 16.1 years. The study reported that 58.8% had primary amenorrhea, 23.5% had secondary amenorrhea, and 17.6% had oligomenorrhea [4].

Idiopathic early ovarian failure may be caused by unknown mechanisms that affect the apoptotic rate of oocytes. This can lead to fewer oocytes in the ovaries at birth or accelerated atresia. Diagnosis is usually delayed as there is no consensus for diagnosing primary ovarian failure in adolescents. It is important to counsel the patient in relation to their present condition and the risk associated with it [5].

In our case, the age of presentation was at 15 years, and the presenting complaint was non-development of secondary sexual characteristics along with primary amenorrhea. In our case, the patient was already on hormonal treatment. As in cases where the uterus size is small, hormonal treatment to increase the size of the uterus would delay the treatment. The patient conceived with IVF in her first attempt with donor oocytes at the age of 24 years with IVF.

Pregnancy, polycystic ovarian syndrome, hypothalamic amenorrhea, thyroid abnormalities, hyperprolactinemia, and primary ovarian failure are possible causes of amenorrhea in young women [6].

The parents showed different emotions of empathy compared to patients who were in deep sadness. Impaired self-esteem and emotional distress persisted after the diagnosis of primary ovarian insufficiency, and psychological counseling was offered [7].

In cases of premature ovarian failure because of occasional spontaneous resumption of ovarian function, there are 5%-10% chances of natural conception [8]. However, the resumption of fertility is unpredictable, and there is no treatment to improve spontaneous conception.

Women with premature ovarian failure are less likely to conceive naturally and mostly need assisted reproductive technology (ART) for conception [9]. Factors contributing to IVF failure include reduced ovarian reserve, increased maternal age, and procedural complications (cystectomy, salpingectomy, and adhesiotomy). The European Society of Human Reproduction and Embryology (ESHRE)'s Reproductive Endocrinology Special Interest Group proposes early screening and treatment of women diagnosed with ovarian failure. The initial evaluation consists of a diagnostic, fundamental evaluation. Hormonal therapy is part of the management.

Hormone replacement therapy involves taking estrogen and progesterone to replace the hormones that the ovaries are no longer producing. This can help manage menopausal symptoms and improve fertility potential. Oocyte donation is a physical and acceptable option for those whose ovarian reserve has already been depleted to a critical level. Gestational surrogacy is an option for carrying a pregnancy on behalf of the lady having premature ovarian failure if the lady is not fit to carry the pregnancy, provided the male partner's gamete is acceptable for IVF/intracytoplasmic sperm injection (ICSI). There are few experimental techniques such as homologous ovarian tissue transplantation, stem cell therapy, or other innovative approaches aimed at restoring ovarian function. Seeking support from therapists or counselors experienced in infertility issues can help manage the emotional toll and guide decision-making. Adopting a healthy lifestyle can promote overall well-being and potentially improve fertility outcomes. This includes maintaining a balanced diet, regular exercise, stress reduction techniques, and avoiding smoking or excessive alcohol consumption.

Premature ovarian insufficiency (POI) affects reproductive health, conception, bone health, cardiovascular system, and psychosexual and neurological functions. It is important to start evaluation early to make a proper diagnosis [10]. The patient and her family members should be counseled regarding the patient's condition on future fertility management. When desired by the patient and her family, referrals to an infertility specialist and a reproductive endocrinologist should be made to discuss available reproductive treatment measures. As in our case, the patient was already evaluated for the absence of secondary sexual characteristics and primary amenorrhea, and the patient reached out to us timely for fertility management.

## Conclusions

Pregnancy and live birth rates largely depend on the female's age at the time of IVF. IVF with donor oocyte is one the most feasible and successful management options for candidates with premature ovarian failure. This case highlights the importance of early diagnosis, treatment, psychological support, and proper guidance regarding fertility so that patients can complete their families timely.
